# 

**Published:** 1874-05

**Authors:** 


### CONTENTS:

### ORIGINAL COMMUNICATIONS.

ARTICLE I.—Diseased Conditions—their Effects upon the Teeth. By Dr. G. V. Black..... 1

ARTICLE II.—Taking Upper Impressions of the Mouth. By Dr. Geo. S. Fouke .............. 16

### SELECTED ARTICLES.

ARTICLE III.—The Advantages of Capping Pulps of Teeth. By Dr. F. E. Howard. ........ 20

ARTICLE IV.—An Unexpected Property of Adhesive Gold. By Thomas Fletcher, F. C. S. 29

ARTICLE V.—Improvements in Dentistry. By Samuel Welchens, D D. S. .................... 32

ARTICLE VI—Root Filling. By C. W. Spalding, D. D S.......................................... 35

ARTICLE VII.—Zinci Oxychloridum. By John Fairbank, M. R. C. S ................................ 37

### EDITORIAL, ETC.

The Diarrhœa of Teething Children.................................................... 40
Replantation of Teeth.................................................................. 41
Can a Person be Anæsthetized During Sleep?........................................ 42

### MONTHLY SUMMARY.

Apparent Death—Recognition by Faradization....................................... 44

Death from Chloroform—Damages...................................................... 45

Treatment of Chloroform Asphyxiation............................................... 45

A Little Studied Cause of Hoarseness................................................. 45

Treatment of Salivation by Atropia.................................................... 46

Cancer Cured by Faith.................................................................. 46

Remedy for Chronic Hoarseness....................................................... 46

Administration of Ether................................................................. 46

The Areca or Betel Nut.................................................................. 47

Laughter as a Medicine.................................................................. 47

Hygiene of Dwellings..................................................................... 48

Guarana for Cure of Headache.......................................................... 48

